# Pharmacokinetic and Pharmacodynamic Factors That Can Affect Sensitivity to Neurotoxic Sequelae in Elderly Individuals

**DOI:** 10.1289/ehp.7568

**Published:** 2005-05-26

**Authors:** Gary Ginsberg, Dale Hattis, Abel Russ, Babasaheb Sonawane

**Affiliations:** 1 Connecticut Department of Public Health, Hartford, Connecticut, USA; 2 Clark University, Center for Technology, Environment and Development, Worcester, Massachusetts, USA; 3 National Center for Environmental Assessment, Research and Development, U.S. Environmental Protection Agency, Washington, DC, USA

**Keywords:** adverse drug reactions, geriatric, metabolism, neurotoxicity, pharmacokinetics, polypharmacy

## Abstract

Early-life exposure to agents that modulate neurologic function can have long-lasting effects well into the geriatric period. Many other factors can affect neurologic function and susceptibility to neurotoxicants in elderly individuals. In this review we highlight pharmacokinetic and pharmacodynamic factors that may increase geriatric susceptibility to these agents. There is a decreasing trend in hepatic metabolizing capacity with advancing years that can affect the ability to clear therapeutic drugs and environmental chemicals. This factor combined with decreased renal clearance causes prolonged retention of numerous drugs in elderly individuals. A geriatric pharmacokinetic database was developed to analyze changes in drug clearance with advancing age. This analysis shows that the half-life of drugs processed by hepatic cytochrome P450 enzymes or via renal elimination is typically 50–75% longer in those older than 65 than in young adults. Liver and kidney diseases are more common in elderly individuals and can further decrease the clearance function of these organs. Polypharmacy, the administration of numerous drugs to a single patient, is very common in elderly individuals and increases the risks for drug interaction and side effects. With advancing age the nervous system undergoes a variety of changes, including neuronal loss, altered neurotransmitter and receptor levels, and decreased adaptability to changes induced by xenobiotics. These changes in the central nervous system can make elderly individuals more susceptible to neurologic dysfunction when confronted with single pharmacologic agents, polypharmacy, or environmental toxicants. The many factors that affect elderly responses to neuroactive agents make environmental risk assessment for this age group a special concern and present a unique challenge.

Early-life exposures to heavy metals, pesticides, and other neurotoxicants can lead to long-lasting and/or delayed effects on the nervous system (Barone et al. 2001; Thiruchelvam et al. 2002). Such effects may accelerate the natural aging process and may be most prominent in elderly individuals (Moser 2001). However, early-life exposures are only one factor that may affect functioning of the nervous system during the geriatric period. The aging process itself has substantial effects on the brain and peripheral nervous system (Masoro and Snyder 2001; Weiss 2000). In addition, exposure of elderly individuals to neuroactive drugs or environmental chemicals can alter central nervous system (CNS) function and lead to a variety of symptoms (Hein et al. 1990; Overstreet 2000; Wozniak et al. 1991). Elderly individuals can have different sensitivity to these agents than younger adults because of pharmacokinetic factors (chemical absorption, distribution, metabolism, excretion) and pharmacodynamic factors that control cellular response to the chemical. Thus, although there has been much focus on how early-life exposures may affect later-life function, this influence needs to be seen within the context of myriad other factors that occur in elderly individuals.

This article is a review of some of the pharmacokinetic and pharmacodynamic differences between younger and older adults that may lead to altered sensitivity to chemical exposure. The review capitalizes upon pharmacokinetic data for therapeutic drugs in elderly individuals to estimate geriatric/younger adult differences in key pathways that can affect the handling of a wide array of xenobiotics. As shown in Figure 1, therapeutic treatment of illness requires information on both pharmacokinetics and pharmacodynamics to understand interpatient variability and how to titrate dosage for body size, age, or genetic traits. This leads to clinical pharmacokinetic drug trials, which when compiled for numerous drugs can reveal metabolism and clearance differences across age groups (Ginsberg et al. 2002). If sufficient clinical pharmacokinetic data are available, it may be possible to make inferences about the handling of environmental toxicants in a particular age group such as elderly individuals.

Given that it would be unethical to purposely expose a potentially sensitive population to even trace levels of an environmental toxicant, there is a lack of pharmacokinetic (termed “toxicokinetic” when referring to toxicants) data in elderly individuals. The approach of relying on therapeutic drug studies, as described in this review, can help address this gap. In addition a variety of exposure and pharmacodynamic factors that can lead to adverse drug reactions (ADRs) in elderly individuals are reviewed to help understand occurrences of drug-related neurologic dysfunction in this age group.

## Basic Features of Aging That Can Affect Response to Xenobiotics

Most of us have witnessed the relatively large interindividual variability in the rate of aging that exists in the human population. This variability in the rate of aging can increase intersubject variability in response to xenobiotics compared with younger adults. Although it is clear that biologic age is more relevant than chronologic age, it is not always easy to obtain information showing the biologic age of an organ system. Fortunately, this is feasible for key pharmacokinetic factors such as hepatic metabolism and urinary clearance because these pathways are often determinants of clinical pharmacokinetic studies. Such indices are used in the titration of drug dosage to help achieve optimal activity and minimal side effects in elderly individuals who use drugs with a narrow therapeutic index (Cutler and Narang 1984; Tranchand et al. 2003).

“Normal” aging of organs and systems leads to diminished function in many areas, from decreased neuromuscular strength and reaction time to losses in memory and cognition (Masoro and Snyder 2001; Weiss 2000). These deficits can be accentuated under the influence of physiologic or chemical stressors due to the loss of functional reserve in aging, leading to the manifestation of a clinical disease process or ADR. For example, defenses against oxidant stress appear to decline with age, as seen in *in vitro* studies in which the basal rate of lipid peroxidation increased and the ability to scavenge oxygen radicals decreased with replicative age of the cell cultures (Sitte et al. 2001). Increasing age has been associated with increasing levels of lipid peroxidation as measured by pentane exhalation in human subjects (Zarling et al. 1993). Although antioxidant levels in the frontal lobes of the brain have been reported to decrease in an age-related fashion (Craft et al. 2004), blood antioxidant levels and enzymes may not be lower in older subjects unless there is poor nutrition or active disease processes (Vogel et al. 1997; Wouters-Wesseling et al. 2003; Zarling et al. 1993).

Another example of decreased function in elderly individuals is impaired clearance function in both the liver and kidney, leading to a greater potential for ADR from drug overdose (Muhlberg and Platt 1999; Sotaniemi et al. 1997). The effects of these declines in organ function on the pharmacokinetics of therapeutic drugs and toxicants are discussed in more detail below.

Superimposed on these “normal” age-related decreases in function are disease-related changes in organ structure and function. Diseases of the liver and kidney are more common and typically more advanced in elderly individuals than in younger age groups, leading to a greater likelihood for disease-related decreases in the pharmacokinetic processing of drugs or environmental chemicals (Lam et al. 1997; Regev and Schiff 2001). Disease in key clearance organs can increase intersubject variability in drug response and thus provide further rationale for careful dose titration in elderly individuals.

Another major factor that can affect an elderly subject’s response to administered drugs is the potential for drug–drug or drug–environmental chemical interactions due to polypharmacy. Polypharmacy is a general term describing the use of multiple drugs, often to address different conditions, in a single patient. The number of drugs prescribed can be especially high when certain drugs are used to combat the side effects of the primary drugs given to treat the disease. Polypharmacy is prevalent in elderly individuals because of the greater number of chronic diseases that manifest with increasing age and the greater reliance upon pharmacologic treatment options in this age group (Routledge et al. 2004). Polypharmacy can stress pharmacokinetic and pharmacodynamic systems that are already at a reduced level of function due to normal aging processes and the loss of functional reserve.

## Physiologic Changes That Can Affect Pharmacokinetic Function during Aging

**Figure 2:**
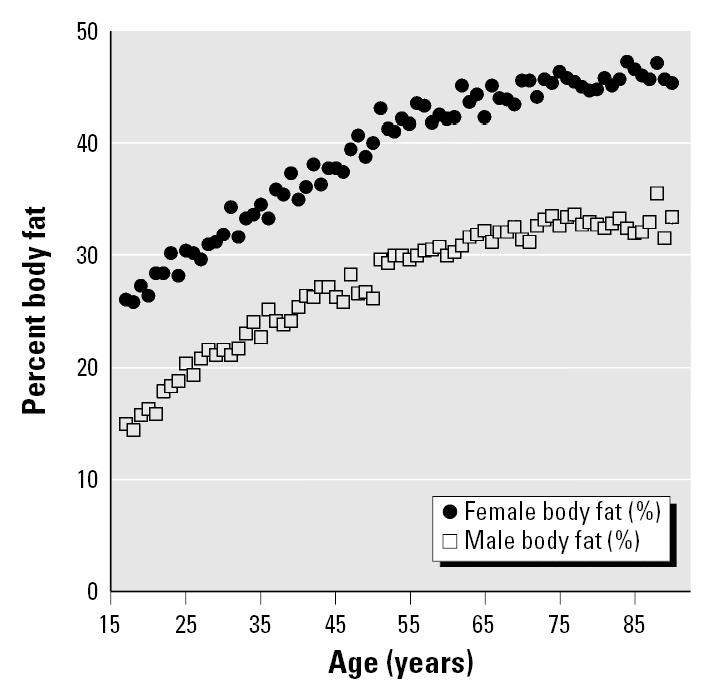
Average percent body fat versus age in men and women: estimates from the National Health and Nutrition Examination Survey III body mass index data using the formulas of Lean et al. (1996).

Body composition changes with advancing age as the percentage of muscle mass and body water decline by as much as 25% in women by age 70 (Masoro and Snyder 2001). These decreases occur while body lipid is increasing, with this compartment rising to > 40% of body weight in elderly women and to > 30% in elderly men (Figure 2).

These changes can be expected to cause a larger volume of distribution and longer half-life of lipophilic chemicals because of their increased sequestration in fat, a tissue that is not involved in body clearance. This has been suggested for dioxin-exposed Vietnam War veterans in whom sequential analysis over 15 years showed that those with higher body lipid had slower dioxin clearance (Michalek and Tripathi 1999). Lipid-soluble drugs have slower clearance in elderly individuals, as shown for certain benzodiazepine antianxiety drugs. For example, diazepam’s volume of distribution and half-life are greater in elderly individuals, which is a function of both slower metabolic oxidation and its high lipid solubility (Barclay 1985; Cutler and Narang 1984; Greenblatt et al. 1983). Diazepam’s prolonged half-life has led to its diminished use in elderly individuals, with replacement anxiolytics such as lorazepam preferred because of more rapid pharmacokinetics.

Another factor that affects drug distribution is the decreased plasma protein binding capacity in elderly individuals. This decrease is 15–25% and has been attributed to increased proteinuria (loss of serum proteins to urine due to changes in renal function) rather than to decreased plasma protein synthesis by the liver (Schmucker 2001). This nonspecific loss of plasma proteins can lead to higher free drug plasma concentration that for highly bound drugs and environmental chemicals can lead not only to a greater percentage available for uptake into target organs but also to a greater percentage available to clearance organs for metabolism or renal elimination (Masoro and Snyder 2001). The reduced protein binding capacity may lead to a greater potential for drug–drug interaction via competitive displacement from the limited number of serum binding sites (Girgis and Brooks 1994).

### Aging and hepatic clearance.

Liver function is generally considered to be maintained fairly well into old age. Several liver function tests such as serum albumin, bilirubin, cholesterol, and alkaline phosphatase are not markedly different in 90-year-olds compared with young adults (Schmucker 2001). However, liver size and hepatic blood flow are diminished 25–35% in elderly individuals, and there is also a decrease in bile flow (LeCouteur and McLean 1998; Zeeh and Platt 2002). Histopathologic examination of livers of aged individuals reveals an accumulation of lipofuscin, a brown pigmented waste product that is formed from the oxidation of lipids and proteins (Anantharaju et al. 2002). This evidence suggests an increase in hepatic lipid peroxidation and/or a decreased capacity to remove this waste product in the liver at advanced age. Cell culture studies demonstrate the increased accumulation of lipofuscin in cells undergoing oxidative stress and with increasing replicative age, supporting the concept that senescent cells have diminished defenses against oxidative stress (Sitte et al. 2001).

**Figure 3:**
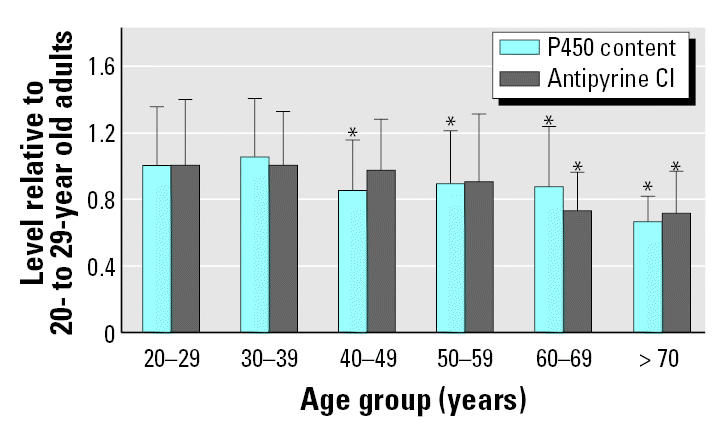
Decline in hepatic CYP function with age. Cl, clearance. Data are mean ± SD. Adapted from Sotaniemi et al. (1997). *p < 0.01.

The specific content of cytochrome P450 (CYP) enzymes in the liver also diminishes during aging. A large liver bank consisting of 226 biopsy specimens of similar histopathologic condition was probed for total CYP content (nmol CYP/gram liver), and antipyrine clearance was measured *in vivo* in the same subjects (Sotaniemi et al. 1997). Antipyrine is a non-specific CYP substrate and thus is a general index of hepatic CYP function. As shown in Figure 3, when the data were broken into age decade of donor, there is a decrease beginning at 40–49 years of age, with further decreases in the 60s and 70s. The maximal decline relative to the youngest age group is 50% for hepatic CYP content and 30% for antipyrine clearance. This is consistent with other evidence (Schmucker 2001) and suggests that multiple factors such as less blood flow to the liver, smaller liver mass, and decreased specific content of CYPs may combine in elderly individuals to decrease hepatic clearance of drugs.

A therapeutic drug pharmacokinetic data set in elderly individuals was developed on the basis of published studies to further evaluate *in vivo* drug clearance function with increasing age (Hattis and Russ 2003). Data for 46 drugs encompassing a variety of clearance pathways including CYP-mediated phase I metabolism, phase II conjugation, and renal excretion as parent drug were included in the database (Table 1). The database includes results for more than 4,500 subjects, reported as either individual or group mean data for drug clearance, half-life, volume of distribution or area under the curve (AUC) of drug concentration in blood × time after dosing (Tables 2, 3). Subjects were grouped in 5-year age increments following the reference age group, 18–24 years of age. Most age groups are represented by more than 100 subjects, with the minimum number being 45 subjects in the over-85 age group.

Analysis of changes in drug clearance across multiple age groups and drugs was performed using multiple regression techniques as described in a previous analysis of a pediatric pharmacokinetic data set (Ginsberg et al. 2002). Separate regression coefficients were derived for each age group and end point [half-life, clearance, AUC, volume of distribution (V_d_)], with the regression coefficient representing the age group:young adult ratio of parameter values.

**Figure 4:**
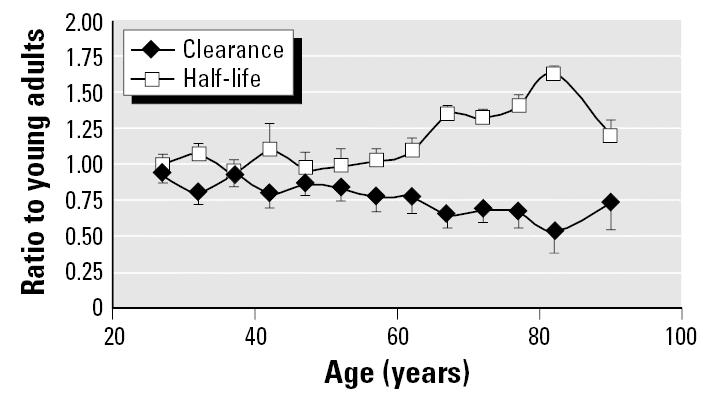
Change in drug clearance and half-life with increasing age across all drugs in pharmacokinetic database. Data points are geometric mean ± SE.

Figure 4 shows geometric mean drug clearance and half-life results across all drugs in the database, with results expressed as the ratio of clearance or half-life in the particular age group compared with the reference, young adult group. The figure shows that drug clearance and half-life remain similar to the young adult level through 60 years of age, but that greater half-life and slower clearance are seen in the subsequent age groups. The maximal change is in the 80- to 84-year-old group, with half-life increased by 60% and clearance decreased by 50%. All age groups older than 65 were significantly different than the reference group except the over-85 age group, for which the results are somewhat closer to the reference group. However, the data for the over-85 age group are the least robust, as they are based upon the smallest data set.

**Figure 5:**
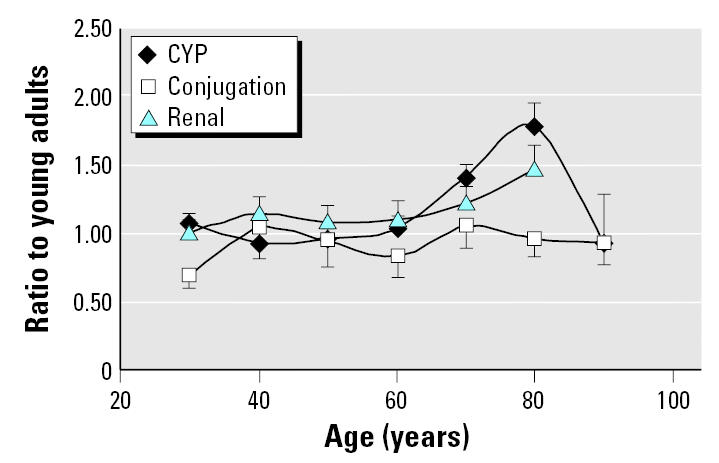
Comparison of half-life across age groups for different clearance mechanisms. Data points are geometric mean ± SE.

Figure 5 provides an analysis of across-age differences in half-life for drug groupings based on broad categories of clearance mechanism: CYP-mediated phase I metabolism, phase II conjugation without prior oxidation, and renal excretion. The results for CYP and renal elimination are consistent with the overall trend shown in Figure 4, with significant increases in half-life in the over-65 age group. Once again, the over-85 age group does not show a difference from the reference group, likely because of the small sample size available for each pathway. It is also possible that particularly robust individuals tend to be entered into clinical pharmacokinetic studies at this age. Phase II conjugation shows no evidence of decline with advancing age.

These results indicate that the physiologic changes in liver that occur during senescence translate into significantly less drug clearance in elderly individuals older than 65. This elderly/young adult pharmacokinetic difference is 50–75% for drugs cleared by a variety of CYPs or via renal excretion. The basis for the increase in half-life for drugs cleared by renal elimination is discussed further in the next section. Recognition of decreased drug clearance in elderly individuals has led to dose adjustment for numerous drugs to avoid overdose.

**Figure 6:**
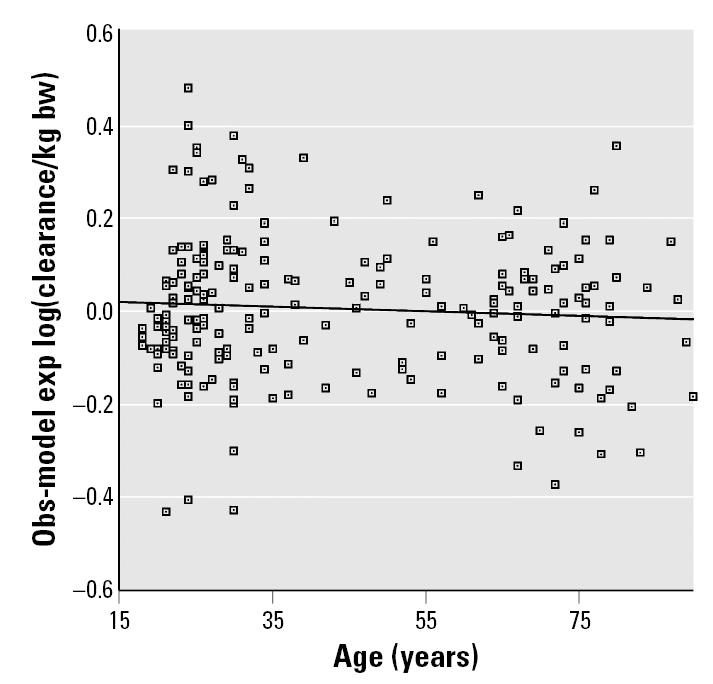
Scatter plot of observed (Obs) versus model expected (exp) log(clearance/kg bw). bw, body weight. *y* = –0.0269 to 0.00050*x*; *R*^2^ = 0.005.

The geriatric pharmacokinetic database was also used to assess interindividual variability in drug clearance in elderly age groups compared with that in younger adults (Hattis and Russ 2003). Figure 6 is a scatter diagram depicting the variability in drug clearance for individual subjects. Data are shown as the difference in clearance from that expected based upon the overall data set regression. The figure shows substantial interindividual variability in each age group, but when statistically analyzed, there was no evidence for an increase in variability in older adults. It is unclear whether and to what degree enrollment in these various pharmacokinetic drug trials is constrained in terms of concurrent illnesses or drug therapy. Such enrollment constraints would tend to limit the degree of variability seen in the subjects in these studies compared with that in the general population, especially for older age groups where these factors are more prevalent.

### Aging and renal clearance.

The aging process has deleterious effects on renal function, with decreases in renal weight and number of glomeruli in subjects older than 50. In addition, the basement membrane thickens and the number of schlerotic glomeruli increases. These changes can occur because of normal aging but are accelerated by the onset of disease processes (Muhlberg and Platt 1999). Decreases in renal blood flow have been approximated at 10% per decade beginning after the fourth decade (Muhlberg and Platt 1999); these decreases can lead to glomerular ischemia in elderly individuals. These factors combine to decrease glomerular filtration rate with data from 256 geriatric patients pooled across 17 pharmacokinetic studies indicating a steady decline in creatinine clearance in subjects older than 60 (Muhlberg and Platt 1999). Subjects who had the lowest creatinine clearance were also the ones in which drug plasma levels attained potentially toxic levels. Cisplatin and other drugs that are cleared via renal excretion are dosed in elderly individuals on the basis of renal clearance function to ensure that toxic levels do not build up in blood (Dollery 1999; Legha 1990). Thus, titration of drug dosage is particularly important for drugs that are cleared by or can damage the kidneys. For example, acyclovir-induced renal failure and concomitant neurotoxicity are more likely to occur in those whose creatinine clearance rate is already somewhat diminished (Johnson et al. 1994).

## Liver and Kidney Disease in Elderly Individuals

The preceding discussion focused on subjects with no overt liver or kidney disease and so could be described as the natural course of aging of hepatic and renal function. However, disease-induced decrements in liver and kidney clearance of xenobiotics are an important overriding factor leading to altered pharmacokinetics and possibly increased variability in elderly individuals.

Although some organ diseases occur at other ages as well, their prevalence and severity are generally greater in elderly individuals because of more time for accumulation of damage and decreasing functional reserve (Mahon and James 1994). The senescent liver has reduced regenerative capacity such that recovery is impaired in response to viral or toxic insult or disease process (Regev and Schiff 2001). Diseases such as alcohol-induced cirrhosis, viral hepatitis–induced cirrhosis, hepatocellular carcinoma, diabetic-associated chronic liver disease, and biliary cirrhosis are more prevalent in the elderly and are associated with replacement of functional tissue with fibrotic or tumorous tissue or fatty lesions (Adler et al. 2002; Amarapurkar and Das 2002; Jansen 2002; Van Dam and Zeldis 1990). These changes can affect hepatic blood flow and the mass of metabolizing tissue available for drug clearance.

A variety of drugs can induce hepatotoxicity with the possibility that reduced cellular defenses and reserve capacity could make the elderly individuals’ liver more susceptible to these drugs. For example, serum transaminases were used as an index of hepatotoxicity from combined isoniazid/rifampin therapy in pulmonary tuberculosis patients (Van Den Brande et al. 1995). Before antituberculosis treatment, serum transaminase levels were similar in a group of 67 young patients (mean age, 38.6 years) and 64 elderly patients (mean age, 71.2 years); subjects with pre-existing hepatic disease were eliminated from the study. During the course of treatment, serum transaminase levels in the young group increased approximately 2-fold, whereas a statistically greater increase of 4- to 5-fold was seen in the elderly group. Another study found that the incidence and severity of hepatic side effects of antituberculosis therapy were high in the elderly individuals, especially those with pre-existing hepatitis (Schaberg et al. 1996).

Sensitivity to drug-induced hepatotoxicity is likely dependent on a number of factors, including capability of hepatocytes to activate and detoxify the particular drug or chemical. Thus, one cannot generalize that elderly individuals will always be more sensitive to the effects of hepatotoxicants. However, where drug-induced hepatotoxicity does occur in elderly individuals, as exemplified by the anti-tuberculosis drugs, one can expect there to be reduced hepatic extraction of other xenobiotics leading to the potential for toxic drug interactions.

Another chemical-induced hepatic effect that may be more prevalent in elderly individuals is liver cancer. This may be counterintuitive from the perspective that carcinogen exposure at a late stage in life leaves little time for expression of the chemical-induced genetic or biochemical change. However, a number of studies have shown a greater increase in liver tumors in response to promotional carcinogens (phenobarbital, peroxisome proliferators) when dosing was initiated in aged as opposed to young adult rats (Cattley et al. 1991; Kraupp-Grasl et al. 1991; Ward et al. 1988). The mechanism for this age-related vulnerability may be that promotors act on clones of cells that already have been transformed by an initiating (typically genotoxic) carcinogen. The number of initiated clones is believed to increase throughout the life span as a result of cumulative exposure to initiators, thus giving promotors a larger population of cells to act on in elderly individuals (Cattley et al. 1991).

Renal disease is also more common in elderly individuals. The chronic effects of hypertension and type 2 diabetes on the renal vasculature lead to renal diseases involving nephroschlerosis, atherosclerosis, and athero-embolism (Gomez et al. 1998; Mulder and Hillen 2001; Ritz and Tarng 2001). Nephropathy and reduced renal blood flow can progress to end-stage renal disease and the need for dialysis. Even before this point, renal disease can lead to decreases in renal blood flow, glomerular filtration, and tubular transport processes (Lam et al. 1997). This can be expected to decrease the clearance of water-soluble drugs and drug metabolites that rely upon glomerular filtration or tubular secretion mechanisms for entry into urine. Plasma protein binding can also be further diminished as greater amounts of these proteins are lost from blood into urine. Given that the effects of renal disease on the clearance of drugs are combined with the normal aging decrement in renal function, titration of drug dosage to renal function as estimated by creatinine clearance is especially important in elderly patients (Lam et al. 1997).

A variety of drugs can produce renal side effects, such as nonsteroidal anti-inflammatory agents, aminoglycoside antibiotics, amphotericin B, and acyclovir. The elderly are generally more sensitive to the renal toxicity caused by these agents because their elimination is already compromised because of aging-related decreased renal function, leading to greater concentrations in blood and kidney (Johnson et al. 1994; Muhlberg and Platt 1999). This drug-induced worsening of renal function can lead to interactions between these and co-administered drugs that also rely on renal elimination.

## Polypharmacy

As mentioned above, certain drugs can affect pharmacokinetic function by producing toxic side effects in the liver or kidney to which elderly individuals may be more susceptible (Girgis and Brooks 1994; Larrey 2002; Muhlberg and Platt 1999). Additionally, a number of pharmacokinetic mechanisms can lead to drug–drug interaction to which elderly individuals may be more susceptible, for example, competition for serum protein binding sites or for metabolic or renal elimination pathways (Seymour and Routledge 1998). One example of this involves the selective serotonin reuptake inhibitors (SSRIs), drugs that are often prescribed to treat depression (Hemeryck and Belpaire 2002). Fluoxetine and paroxetine are SSRIs that are potent inhibitors of CYP2D6 and thus can prolong the half-life of numerous drugs. This is of concern because of the long half-life of these SSRI agents, particularly in elderly individuals, such that drug interactions are possible for weeks after SSRI treatment cessation (Bourin et al. 2001). For this reason, other SSRI agents are preferred in elderly individuals (Spina and Scordo 2002).

**Figure 7:**
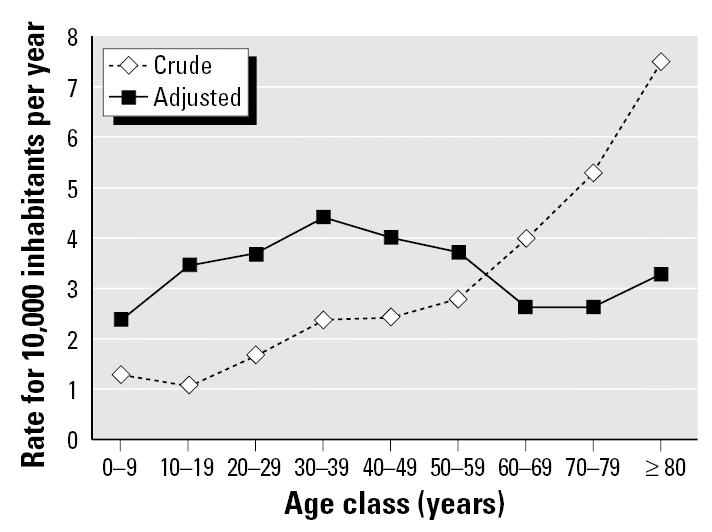
Age-specific rates of adverse drug reactions before and after adjusting for drug consumption. Modified from Begaud et al. (2002).

Although specific drugs may be risk factors for side effects and drug interaction in elderly individuals, the most prevalent factor for the increase in ADR in this age group may be polypharmacy. The elderly consume a disproportionate quantity of drugs, with those older than 65 taking, on average, two to six prescribed and one to three nonprescription drugs at any one time (Routledge et al. 2004). Analysis of French national statistics of reported ADRs and medication use found the expected increase in ADRs with advancing age beyond 55 years (Begaud et al. 2002). However, the increase was not apparent when the data were adjusted for the number of drugs consumed per individual in each age group (Figure 7). This finding agrees with a careful analysis of ADRs, prescription drug administration, age, and other factors in a multicenter clinical study in Italy (Carbonin et al. 1991). The doubling of ADRs between those younger than 50 versus those older than 70 was significantly related to taking numerous drugs, having numerous medical conditions, and longer hospital stays, but there was no independent correlation with age. It thus appears that the cumulative effect of taking numerous drugs can challenge normal physiologic and metabolic functions at any age, but this problem is most acute in elderly individuals because polypharmacy is so prevalent at this life stage.

## Pharmacodynamic Aspects of Sensitivity to Neurotoxic Agents in the Elderly

In addition to the pharmacokinetic and polypharmacy factors described above, there are numerous pharmacodynamic factors in the CNS of elderly subjects that may affect sensitivity to neuroactive agents. Changes in central cholinergic pathways, including decreased number of brain acetylcholine postsynaptic receptors, have been demonstrated in rodent models of aging and may contribute to the progressive decline in memory and cognition in elderly individuals (Pedigo et al. 1984). Aged animals and humans also experience a decline in a number of other biochemical parameters that are critical to central cholinergic transmission. The activity of choline acetyl-transferase, an enzyme in acetylcholine synthesis, is diminished in older subjects. This may be a sign that central cholinergic neurons have diminished integrity (Overstreet 2000). The level of brain acetylcholinesterase also decreases during aging (Bartus et al. 1982). Thus, there are a variety of changes during aging that could affect sensitivity toward anticholinesterase agents (e.g., organophosphate and carbamate insecticides, chemical warfare agents). Some of these changes may tend to counterbalance one another; that is, decreased acetylcholinesterase levels may be offset by decreased levels of acetylcholine synthesis or acetylcholine receptors (Overstreet 2000). This makes it difficult to predict whether elderly individuals will be at higher risk to anticholinesterase agents, and there is insufficient animal data on this to draw conclusions. However, the general diminution of central cholinergic systems suggests decreased functional reserve for adaptation to chemical stresses affecting these systems. Consistent with this is the observation of decreased neurotransmitter receptor plasticity in senescent rats (Pedigo 1994). In these studies, aged rats were unable to up- or down-regulate the numbers of central cholinergic (muscarinic) receptors in response to cholinergic agonists or antagonists (Pedigo 1994). The cholinergic system decrements described above are most pronounced in those elderly with Alzheimer disease.

Another mechanism that may predispose elderly individuals to increased sensitivity to chemical-induced neurotoxicity is deficiency in plasma and tissue esterases. Carboxylesterases (CEs) and A-esterases (AEs) inactivate a wide variety of organophosphate pesticides, either by stoichiometric binding (CE) or via catalytic hydrolysis of the activated, neurotoxic form of the pesticide (AE). Studies in aged (24-month-old) rats demonstrated a markedly increased sensitivity to the acute effects of parathion but not chlorpyrifos (Karanth and Pope 2000). The increased sensitivity to parathion correlated with a 50% reduction of plasma CE in aged rats, whereas levels of AE were unaffected by aging.

Changes in other CNS pathways may predispose elderly individuals to the neurotoxic effects of drugs and environmental chemicals. Aging involves a decline in neuron density in the substantia nigra even in the absence of Parkinson disease (McGeer et al. 1988; Weiss 2000), and this may be related to the increased susceptibility of the dopaminergic system to chemical modulation. This has been seen in aged rats that experienced a greater dopamine depleting effect in the striatal region from acute methamphetamine dosing compared with younger rats (Bowyer et al. 1998). Another agent affecting the central dopaminergic system is 1-methyl-4-phenyl-1,2,3,6-tetrahydropryidine (MPTP). Its acute and residual effects on dopamine levels in the striatum were greatest in 12-month-old mice compared with 7-month-old (intermediate effect) and 23-day-old mice (least effect) (Ali et al. 1993). Additionally, the MPTP-induced effect was rapidly reversible in the younger animals but not in the 1-year-old age group. Similar increases in susceptibility of nigrostriatal neurons in aged mice has been found for paraquat and maneb neurotoxicity (Thiruchelvam et al. 2003).

Elderly humans and rats are more sensitive to the excitotoxic effects of domoic and kainic acids, structural analogs that are chemically related to the excitatory neurotransmitter glutamate (Perl et al. 1990; Teitelbaum et al. 1990; Wozniak et al. 1991). Domoic acid contamination of mussels in Canada in 1987 led to a food poisoning outbreak involving 107 patients, some of whom experienced severe gastrointestinal and neurologic symptoms and pathology of the hippocampus, the main target region of the brain. Acute and protracted neurologic effects were more pronounced in elderly subjects, which led to studies in young, middle-aged, and aged rats with kainic acid (Wozniak et al. 1991). The dose response for seizures was approximately 3 times greater in aged compared with young rats. A number of changes in excitatory and inhibitory pathways accompany aging, including decreases in kainic acid binding in the hippocampus and increased levels of glutamate (Masoro and Snyder 2001). However, the mechanism for increased sensitivity to domoic and kainic acid in elderly individuals has not been uncovered.

These examples point out that aging involves pharmacodynamic changes that can predispose to greater neurotoxic effects compared with earlier life stages. Another factor that may affect CNS function in elderly individuals is the long-term sequelae of earlier exposure to neurotoxic agents. For example, a study of Danish workers found that those whose occupations involved exposure to a mixture of organic solvents had a greater prevalence of memory loss and decreased ability to concentrate relative to a reference group (Hein et al. 1990). This pattern persisted into retirement, where these CNS deficits and an additional finding of increased headache manifested in solvent-exposed, retired workers. Similarly, there is evidence that occupational exposure to heavy metals such as mercury, manganese, aluminum, and lead can lead to long-term decrements in neurologic function because of the irreversible and cumulative effects of these neurotoxicants (Hochberg et al. 1996; Payton et al. 1998; Rifat et al. 1990; Weiss 2000).

## Summary

Numerous types of exposures, either singly or in cumulative fashion, may lead to increased risk for neurologic deficits in elderly individuals. Early-life exposures and workplace exposures may have long-lasting effects on CNS function that may become more significant as the normal aging process decreases functional reserve. Therapeutic drugs are often a risk factor because many are neuroactive, and susceptibility in elderly individuals is increased because of decreased clearance or pharmacodynamic factors that increase vulnerability. The large number of drugs prescribed to an elderly patient at a single time (polypharmacy) and the existence of underlying pathology of the major clearance organs or CNS can increase the likelihood for neurotoxicity. These factors combine to increase the possibility that elderly individuals generally are more susceptible to the neurotoxic effects of environmental chemicals. The extent to which this is true and the possible size of age-related sensitivity differences has not been well studied.

Assessing risks to elderly individuals from neurotoxicant exposure that occurs in early, mid-, or late stages of life is challenging because of the various factors summarized in this article that can alter sensitivity and increase variability in this age group. Risk assessments can be tailored to geriatric populations by adjusting input parameters in standard physiologically based pharmacokinetic (PBPK) models to account for the changes in physiology, metabolism, and renal elimination that are known to take place during aging (Clewell et al. 2002). Such modeling approaches can point out whether the internal dose of a toxicant or its metabolites are likely to be elevated in healthy geriatric subjects and in those whose hepatic or renal function has been compromised by a disease process. This type of approach can also be useful in simulating chemical–chemical interactions that may affect clearance and other pharmacokinetic parameters (Liao et al. 2002), an especially important application given the numerous interactions possible from polypharmacy in elderly individuals. However, PBPK modeling of geriatric polypharmacy will be complex and may benefit from case studies that demonstrate how this approach can improve geriatric risk assessments. Another area needing further study is the relative sensitivity of elderly individuals to neurotoxic agents as affected by pharmacodynamic factors. The increasing emphasis being placed on understanding the responses of elderly individuals to drugs and environmental toxicants should improve our ability to assess risks for this age group in the future.

## Figures and Tables

**Figure 1:**
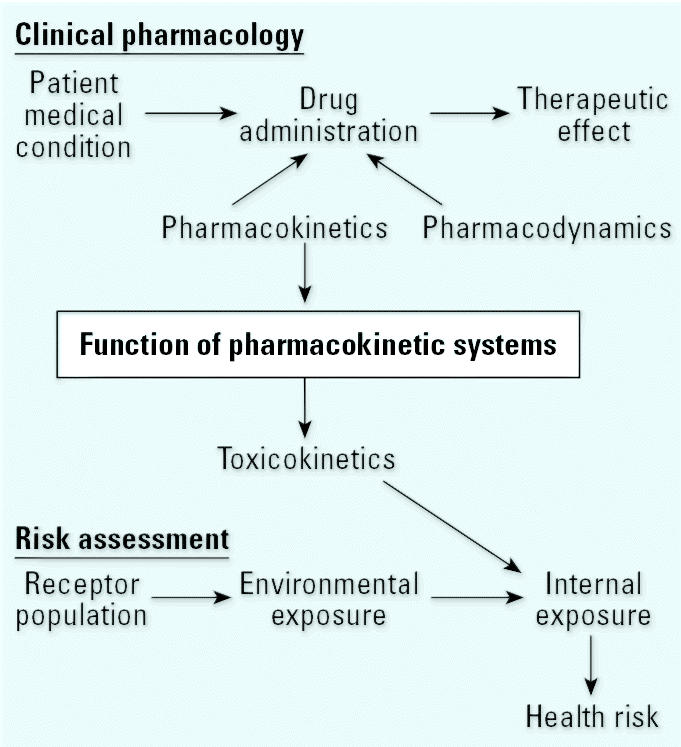
Linkage between clinical pharmacology and environmental risk assessment.

**Table 1:** Major routes of elimination of drugs in the geriatric pharmacokinetic database.

Chemical	Route of elimination	Chemical	Route of elimination
Amikacin	Renal	Lisinopril	Fecal
Amitriptyline	CYP2D6	Lithium	Renal
Ampicillin	Renal	Meperidine	Esterase
Antipyrine	CYP: mixed/unknown	Mephobarbital-*r*	CYP2C19
Atracurium	Esterase	Mephobarbital-*s*	CYP: mixed/unknown
Benazepril	Renal	Mianserin	CYP2D6
Bromazepam	CYP: mixed/unknown	Midazolam	CYP3A or CYP3A4
Bupivicaine	CYP1A2	Nortriptyline	CYP2D6
Chlorpheniramine	CYP2D6	Oxazepam	Conjugation
Chlorzoxazone	CYP2E1	Oxytocin	Other: sulfhydryl reduction and aminopeptidase
Copper	Renal
Diazepam	CYP2C19	Paracetamol	Conjugation
Dicumarol	Unclassified	Pethidine	Esterase
Enalapril	Renal	Phenylbutazone	Conjugation
Enalaprilat	Renal	Phenylpropanolamine	Renal
Erythromycin	CYP3A or 3A4	Piroxicam	CYP2C9
Fentanyl	CYP3A or CYP3A4	Propanolol	CYP2D6
Gentamicin	Renal	Teicoplanin	Renal
Grepafloxacin	Unclassified	Terfenadine	Renal
Ibuprofen	CYP2C9	Theophylline	CYP1A2
Ketanserin	Unclassified	Valproic acid	Conjugation
Ketoprofen	Conjugation	Vancomycin	Renal
Ketorolac	Renal	Viloxazine	Unclassified
Lidocaine	Cyp3A or CYP3A4

**Table 2:** Overview of the database for various parameters.

Parameter	No. of drugs	Individual data points	Data groups^a^	Subjects in data groups (*n*)	Total no. of subjects
AUC	17	163	21	317	480
Clearance	31	210	59	1,003	1,213
Half-life	44	359	84	1,312	1,671
V_d_	26	153	51	996	1,149
Total	46	885	215	—b	—b

V_d_, volume distribution.

a Data sets where pooled (e.g., mean, SD) rather than individual data are available for the indicated parameter.

b Not summed to avoid double counting.

**Table 3:** Summary of the database by predominant modes of elimination.

Route of elimination	No. of drugs	Individual data points	Data groups^a^	Subjects in data groups (*n*)	Total no. of subjects
All CYPs	19	389	126	2,315	2,704
Conjugation	5	122	6	60	182
Other metabolism	4	18	18	114	132
Renal and/or fecal elimination	14	278	51	892	1,170
Unclassified	4	78	14	247	325
Total	46	885	215	3,628	4,513

a Data sets where pooled (e.g., mean, SD) rather than individual data are available for the indicated parameter.
